# Supplementary material for: Cytomegalovirus drives Vδ1+ γδ T cell expansion and clonality in common variable immunodeficiency
Source: Nat Commun. 2024 May 20;15:4286. doi: 10.1038/s41467-024-48527-3 (PMC11106253; doi:10.1038/s41467-024-48527-3)
Supplement: Supplementary file 1 — Supplementary Information [file 41467_2024_48527_MOESM1_ESM.pdf]

Supplementary information for

Cytomegalovirus drives Vδ1$^+$ T cell expansion and clonality in common variable immunodeficiency

Samantha Chan, Benjamin Morgan, Michelle K. Yong, Mai Margetts, Anthony J. Farchione, Erin C. Lucas, Jack Godsell, Nhi Ai Giang, Charlotte A. Slade, Anouk von Borstel, Vanessa L. Bryant & Lauren J. Howson*

*Corresponding author. Email: howson.l@wehi.edu.au (LJH)

The PDF file includes:

Supplementary Figure 1 – T cell gating strategy.
Supplementary Figure 2 – γδ T cells in CVID.
Supplementary Figure 3 – Frequency of Vδ1$^+$ and Vδ2$^+$ γδ T cells producing cytokine.
Supplementary Figure 4 – Sex has minimal impact on γδ T cell frequency.
Supplementary Figure 5 – CVID non-infectious complications do not impact γδ T cell frequency.
Supplementary Figure 6 – CMV serology does not significantly impact γδ T cell subset frequency.
Supplementary Figure 7 – CMV viremia impacts circulating γδ T cells in CVID patients.
Supplementary Figure 8 – Vδ1$^+$ TCRδ clonotype tree plots for all donors.
Supplementary Figure 9 – Vδ2$^+$ TCRδ clonotype tree plots for all donors.
Supplementary Figure 10 – TCRδ chain and TCRγ chain usage for Vδ1$^+$ and Vδ2$^+$ TCR repertoires.
Supplementary Figure 11 – TCRγ repertoire overlap.
Supplementary Figure 12 – TCRδ clonotype tracking for CMV/CVID patient P03.
Supplementary Table 1. CVID patient cohort characteristics.
Supplementary Table 2. Immune T cell counts and frequencies for CVID patients.
Supplementary Table 3. Multivariate analysis of γδ T cell frequencies.
Supplementary Table 4. CMV/CVID patient cohort details.
Supplementary Table 5. Vδ1 clonotypic expansions in CMV/CVID patients.
Supplementary Table 6. Detailed infectious history between sampling for time course analysis.
Supplementary Table 7. Number of cells sorted and sequencing reads for TRG and TRD repertoire analysis.

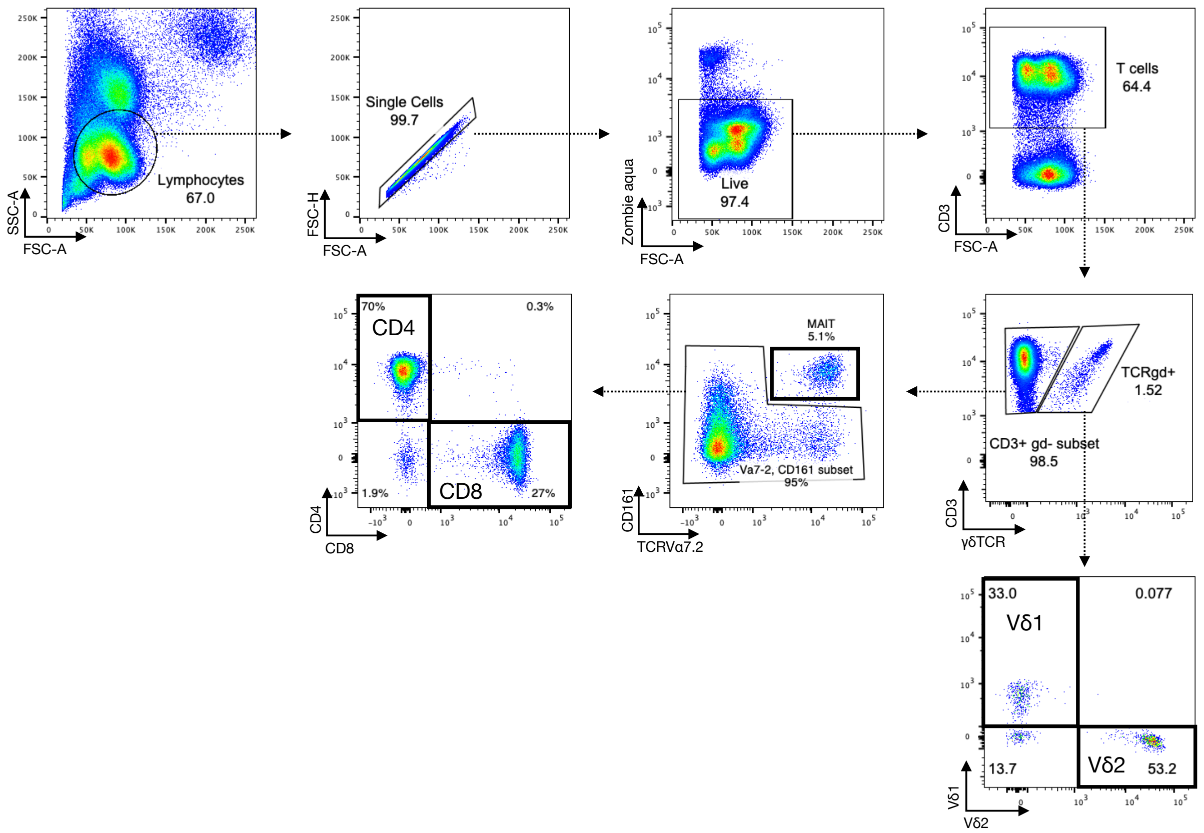

Supplementary Figure 1 – T cell gating and sorting strategy. Plots show the gating strategy of peripheral blood mononuclear cells (PBMC) samples based on forward and side scatter, single cells, live, and $CD3^+$ T cells and then gating on: $CD4^+$ T cells, $CD8^+$ T cells, MAIT cells, and γδ T cells (with $V\delta1^+$ and $V\delta2^+$ subsets). This gating strategy was used for flow cytometry experiments in Figure 1, 2 and 4 and γδ subset sorting strategy for TCR repertoire analysis in Figure 5–7. FSC-A, forward scatter (area); MAIT, mucosal associated invariant T (cell); TCR, T cell receptor; SSC-A, side scatter (area).

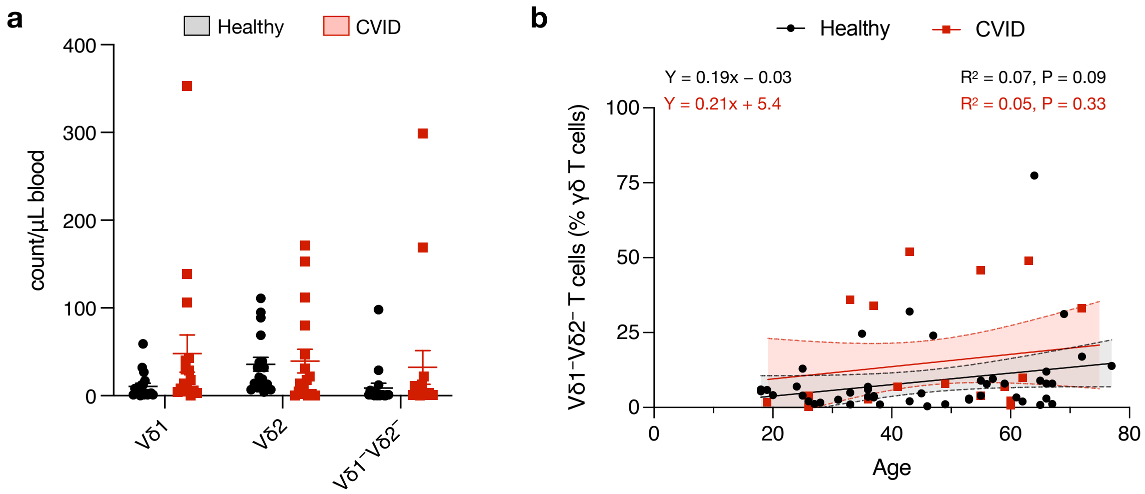

Supplementary Figure 2 – γδ T cells in CVID. PBMCs from healthy individuals and common variable immunodeficiency (CVID) patients were analyzed by flow cytometry. (**a**) Graph showing total counts for γδ T cell subsets (n = 26 healthy, 17 CVID). Each point represents an individual, line is at mean and error bars represent standard error of the mean (SEM). Statistical significance was calculated using two-way analysis of variance (ANOVA) with Sidak's multiple comparison test with single pooled variance. (**b**) Graph of $V\delta1^{-}/V\delta2^{-}$ γδ T cell subset as a frequency of T cells plotted against age (n = 42 healthy, 23 CVID). Line represents simple linear regression with 95% confidence interval of the best fit line shown as dashed line and shaded area. Statistical significance was calculated using simple linear regression (test for non-zero slope) with significance determined when $P < 0.05$ and R^2 indicates goodness of fit.

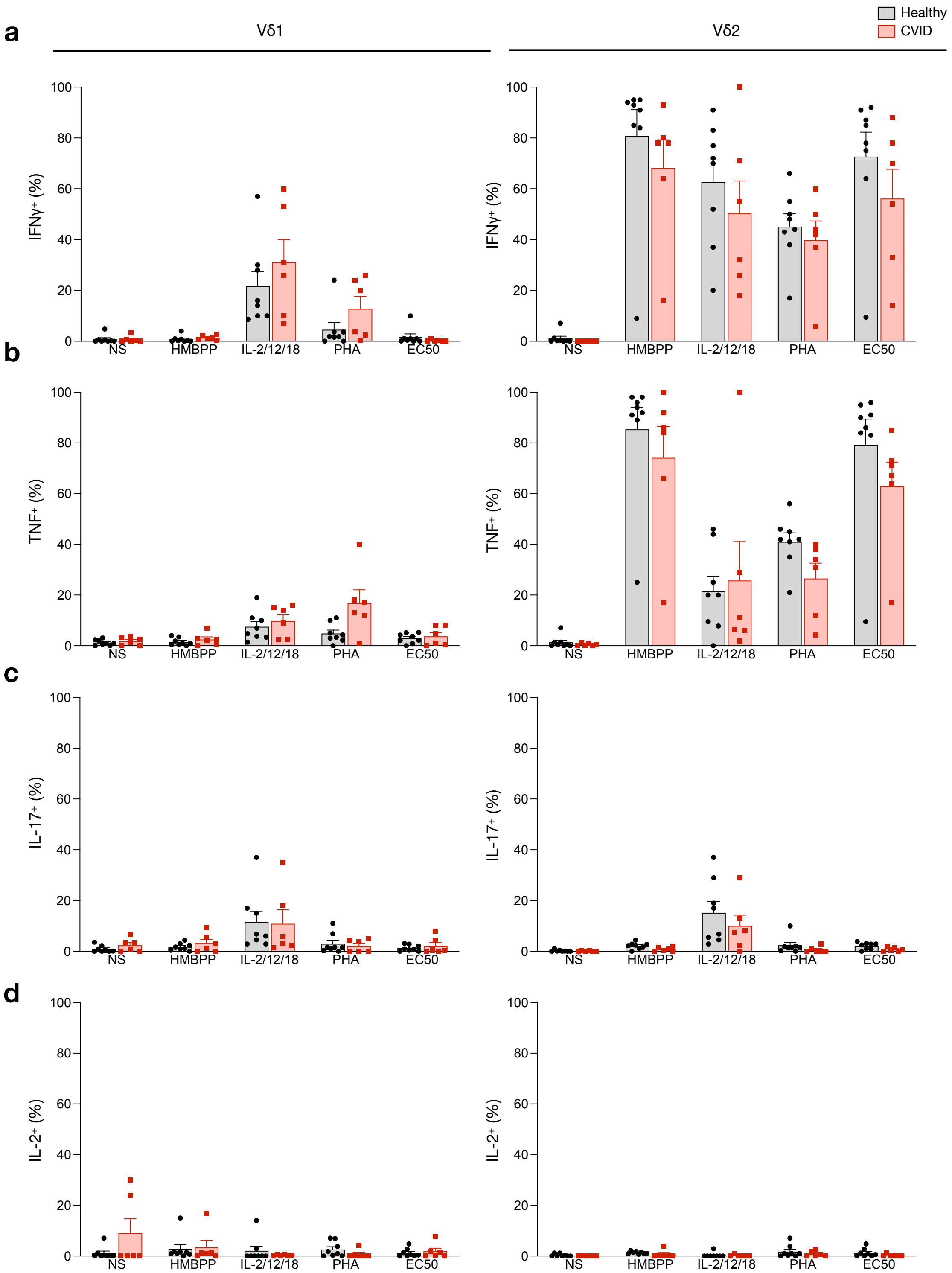

Supplementary Figure 3 – Frequency of Vδ1^{+} and Vδ2^{+} γδ T cells producing cytokine. Graphs shows Vδ1^{+} and Vδ2^{+} γδ T cell response to various stimuli measured by intracellular staining for (**a**) IFNγ, (**b**) TNF, (**c**) IL-17, and (**d**) IL-2 after 18 hr of stimulation of PBMCs in the presence of brefeldin A (BFA) in healthy individuals (n = 8) and CVID patients (n = 5). Each point represents and individual, bars represent the mean and error bars represent SEM. Statistical significance was calculated using two-way ANOVA with Sidak's multiple comparison test. EC, *Escherichia coli,* HMBPP, (E)-4-hydroxy-3-methyl-but-2-enyl pyrophosphate; IFNγ, interferon gamma; IL, interleukin; NS, no stimulation; PHA, phytohemagglutinin; TNF, tissue necrosis factor.

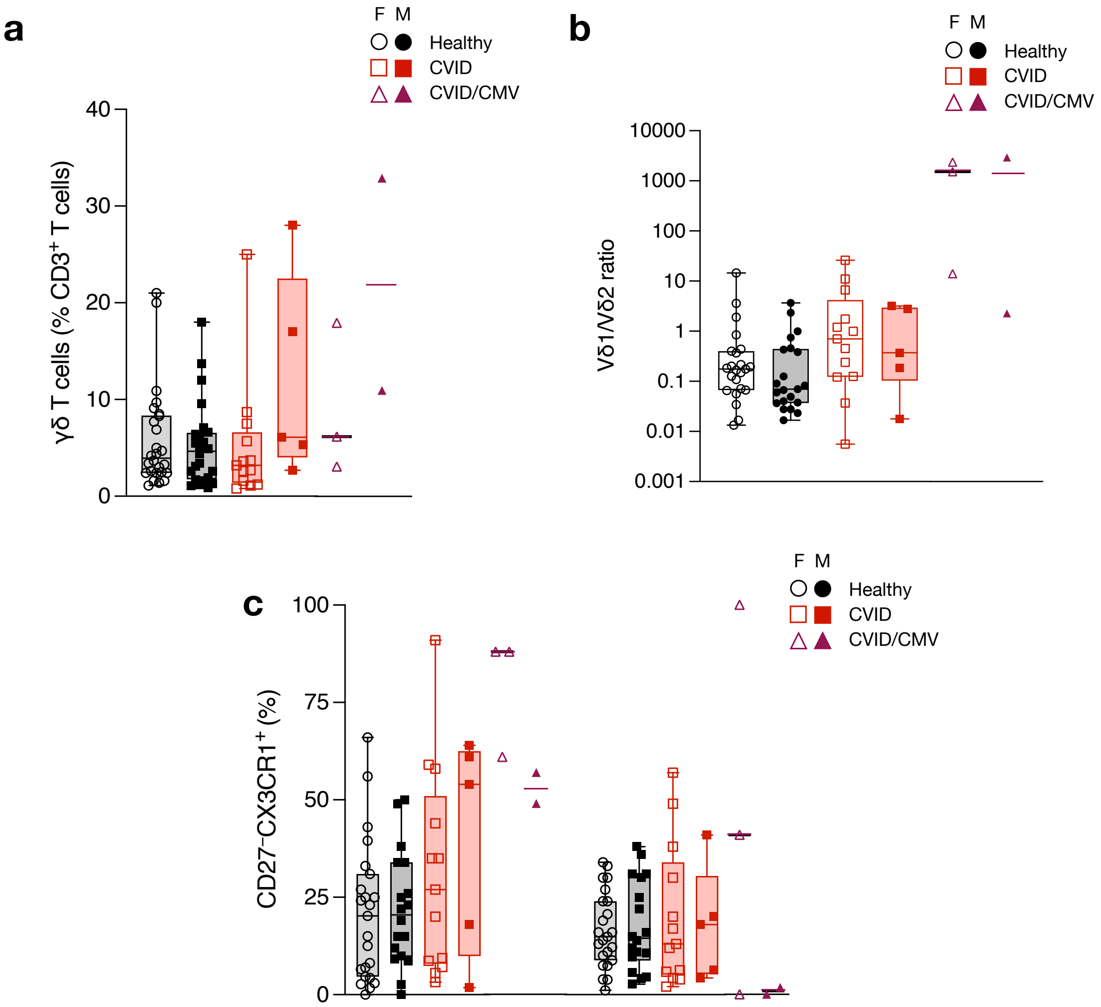

Supplementary Figure 4 – Sex has minimal impact on γδ T cell frequency. The frequency and phenotype of γδ T cells in PBMCs was examined in healthy (n = 24 M, 26 F), CVID (n = 5 M, 13 F) and CMV/CVID (n = 2 M, 3 F) cohorts, separated based on sex. The (**a**) γδ frequency as proportion of T cells, (**b**) the Vδ1/Vδ2 ratio, and (**c**) $CD27^+$ and $CX3CR1^-$ has been graphed. For box and whisker graphs, line is at median, box is upper and lower quartiles, and error bars are minimum and maximum values. Each point represents and individual. Statistical significance was calculated using either one-way ANOVA with Holm-Sidak's multiple comparison test with single pooled variance or two-way ANOVA with Sidak's multiple comparison test with single pooled variance. F, female; M, male.

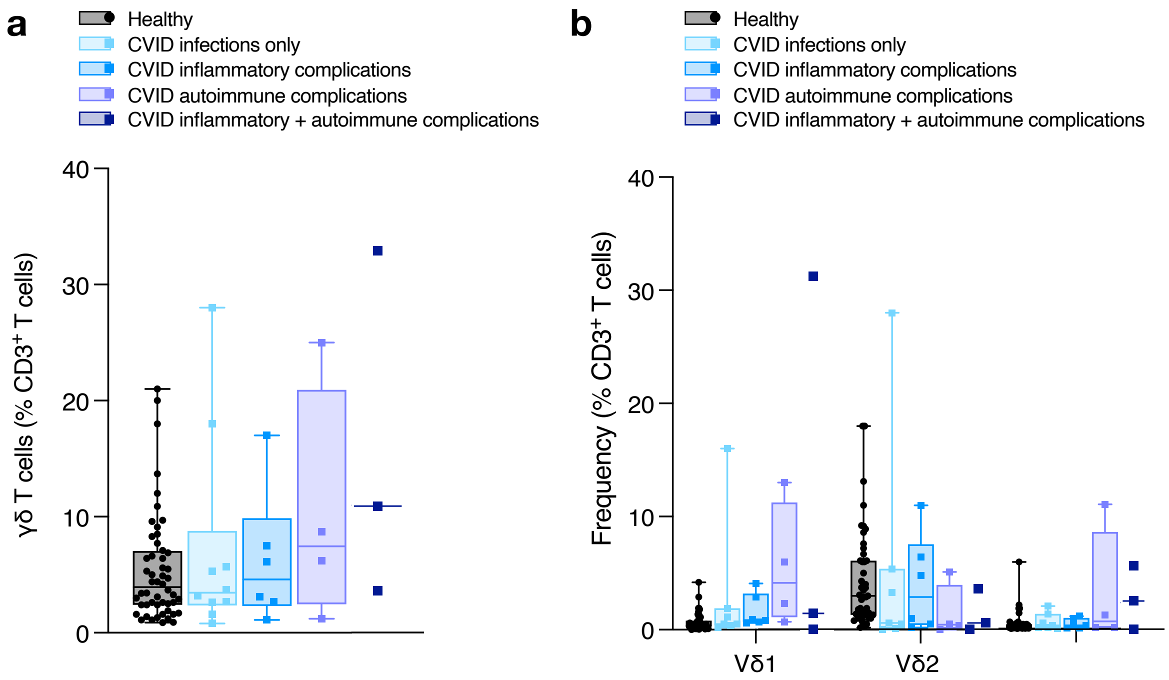

Supplementary Figure 5 – CVID non-infectious complications do not impact γδ T cell frequency. (**a**) The γδ T cell frequency in healthy individuals (n = 50) and CVID patients with either infections only (n = 10), inflammatory complications (n = 6), autoimmune complications (n = 4) or both inflammatory and autoimmune complications (n = 3). (**b**) Vδ1^{+}, Vδ2^{+}, and Vδ1^{-}/Vδ2^{-} γδ T cell subsets as a frequency of T cells in healthy individuals (n = 50) and CVID patients with either infections only (n = 7), inflammatory complications (n = 6), autoimmune complications (n = 4) or both inflammatory and autoimmune complications (n = 3). Each point represents and individual, line is at median, box is upper and lower quartiles, and error bars are minimum and maximum values. Statistical significance was calculated using either one-way ANOVA with Holm-Sidak's multiple comparison test with single pooled variance or two-way ANOVA with Sidak's multiple comparison test with single pooled variance.

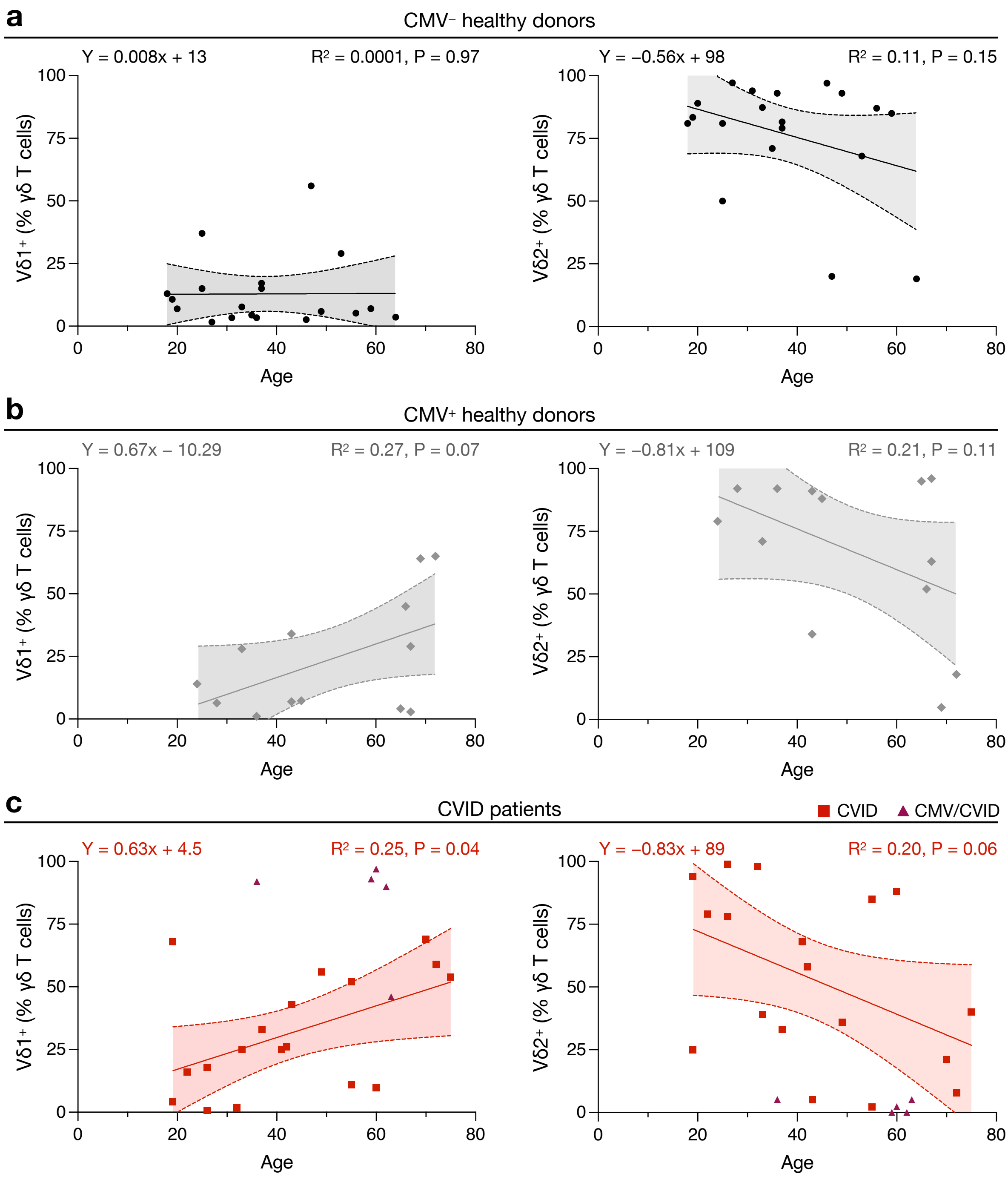

Supplementary Figure 6 – CMV serology does not significantly impact γδ T cell subset frequency. PBMCs from healthy CMV$^-$ individuals (n = 19), healthy CMV$^+$ individuals (n = 15), CVID patients (n = 18), and CMV/CVID patients (n = 5) were analyzed by flow cytometry. Vδ1$^+$ and Vδ2$^+$ cell frequency as a proportion of γδ T cells are plotted against age for (**a**) healthy CMV$^-$, (**b**) healthy CMV$^+$, and (**c**) CVID and CMV/CVID patients. Line represents simple linear regression with 95% confidence interval of the best fit line shown as dashed line. Statistical significance was calculated using simple linear regression (test for non-zero slope) with significance determined when $P < 0.05$ and R^2 indicates goodness of fit.

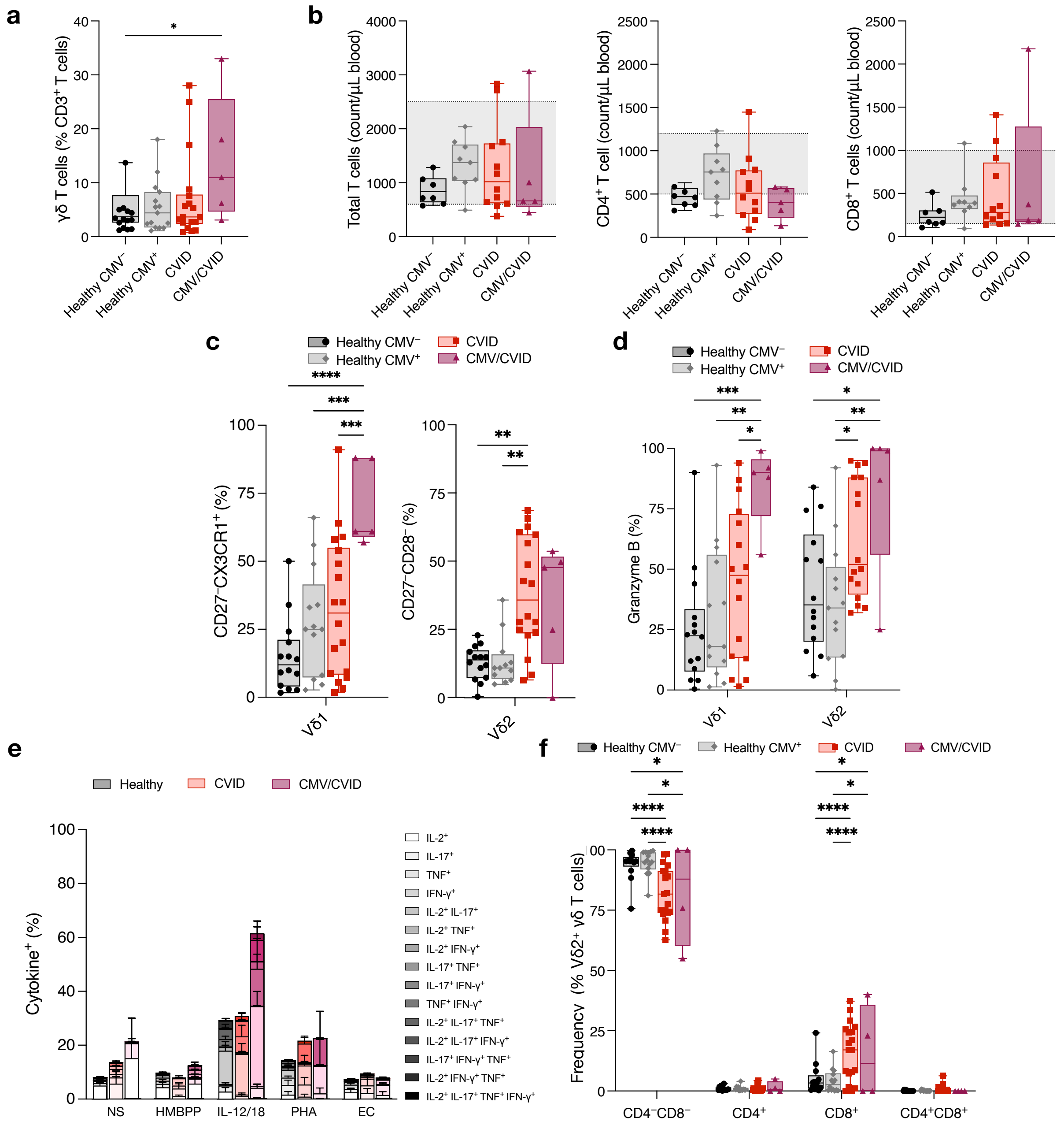

Supplementary Figure 7 – CMV viremia impacts circulating γδ T cells in CVID patients. PBMCs from healthy CMV⁻ individuals, healthy CMV⁺ individuals, CVID patients, and CMV/CVID patients were analyzed by flow cytometry. (**a**) The circulating frequency of γδ T cells (n = 19 CMV⁻, 15 CMV⁺, 18 CVID and 5 CMV/CVID). (**b**) Total counts of T cells, CD4⁺ T cells and CD8⁺ T cells, grey area represents normal healthy range (n = 7 CMV⁻, 9 CMV⁺, 12 CVID and 5 CMV/CVID). The (**c**) CD27⁻ CX3CR1⁺ Vδ1⁺ and CD27⁻ CD28⁻ Vδ2⁺ populations (n = 14 CMV⁻, 12 CMV⁺, 18 CVID and 5 CMV/CVID), and (**d**) granzyme B expression for Vδ1⁺ and Vδ2⁺ γδ T (n = 14 CMV⁻, 13 CMV⁺, 16 CVID and 5 CMV/CVID). (**e**) Stacked graph shows Vδ1⁺ subset polyfunctional cytokine response to various stimuli measured by intracellular staining for IL-2, TNF, IL-17 and IFNγ after 18 hr of stimulation in the presence of BFA (n = 8 healthy, 3 CVID, and 2 CMV/CVID). (**f**) Graph showing coreceptor (CD4/CD8) expression by Vδ2⁺ γδ T cells (n = 13 CMV⁻, 14 CMV⁺, 19 CVID and 4 CMV/CVID). For all graphs, each point represents and individual. For bar graphs, bars represent the mean and error bars represent SEM. For box and whisker graphs, line is at median, box is upper and lower quartiles, and error bars are minimum and maximum values. Statistical

significance was calculated using either one-way ANOVA with Holm-Sidak's multiple comparison test with single pooled variance or two-way ANOVA with Sidak's multiple comparison test with single pooled variance, where $^{*}P < 0.05$, $^{**}P < 0.01$, $^{***}P < 0.001$, $^{****}P < 0.0001$.

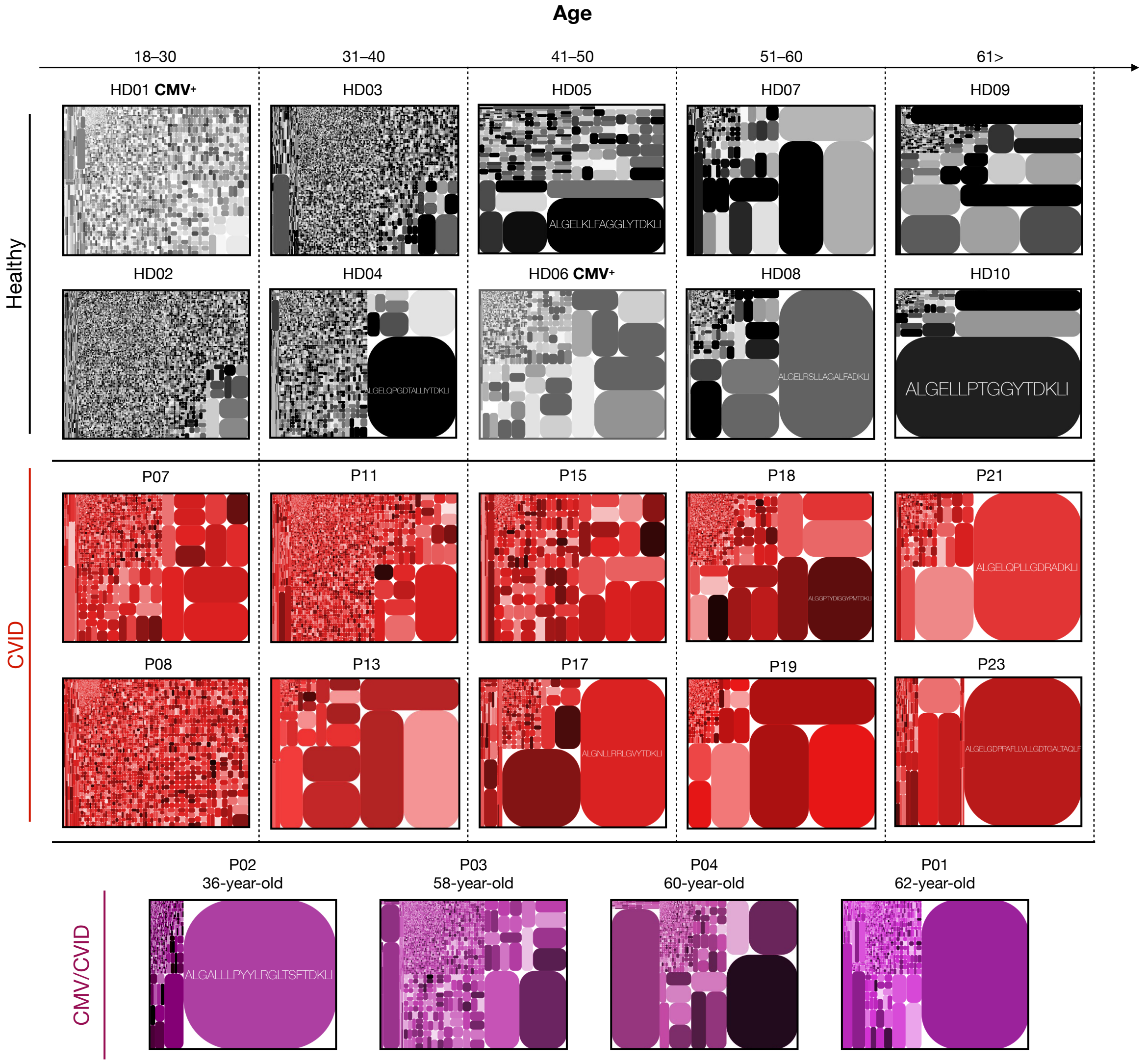

Supplementary Figure 8 – Vδ1⁺ TCRδ clonotype tree plots for all donors. TCRδ clonotype tree plots are shown for Vδ1⁺ γδ T cells populations from healthy individuals (n = 10, 2 are CMV⁺ as indicated), CVID patients (n = 10), and CMV/CVID patients (n = 4). Plots are organized by age category. Tree plots show unique clonotypes (box segments) as a proportion within the total repertoire (size of segment). Color of clonotype boxes do not match between plots or individuals. Major clonotypes have the CDR3δ sequence shown.

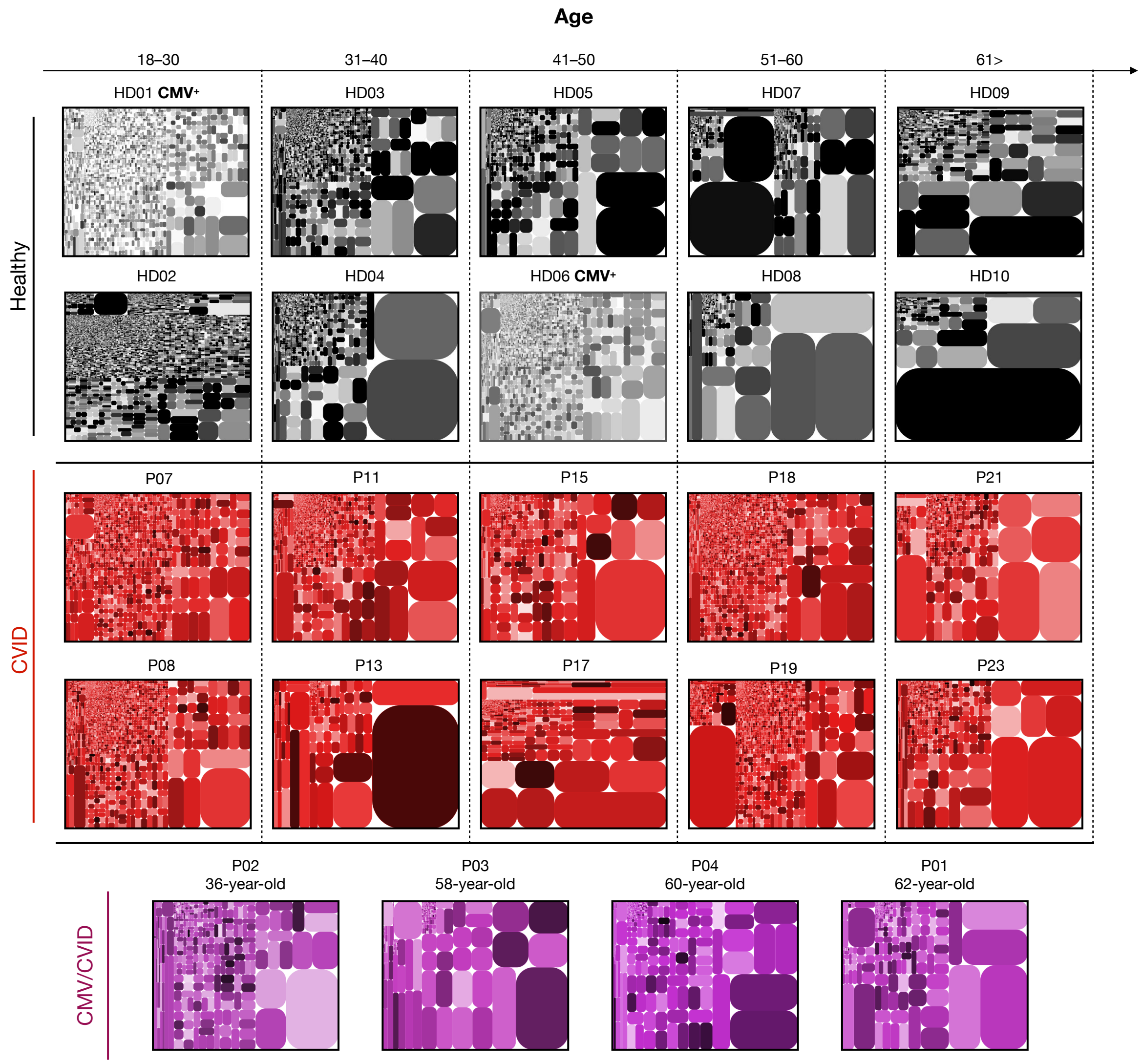

Supplementary Figure 9 – Vδ2⁺ TCRδ clonotype tree plots for all donors. TCRδ clonotype tree plots are shown for Vδ2⁺ γδ T cells populations from healthy individuals (n = 10, 2 are CMV⁺ as indicated), CVID patients (n = 10), and CMV/CVID patients (n = 4). Plots are organized by age category. Tree plots show unique clonotypes (box segments) as a proportion within the total repertoire (size of segment). Color of clonotype boxes do not match between plots or individuals.

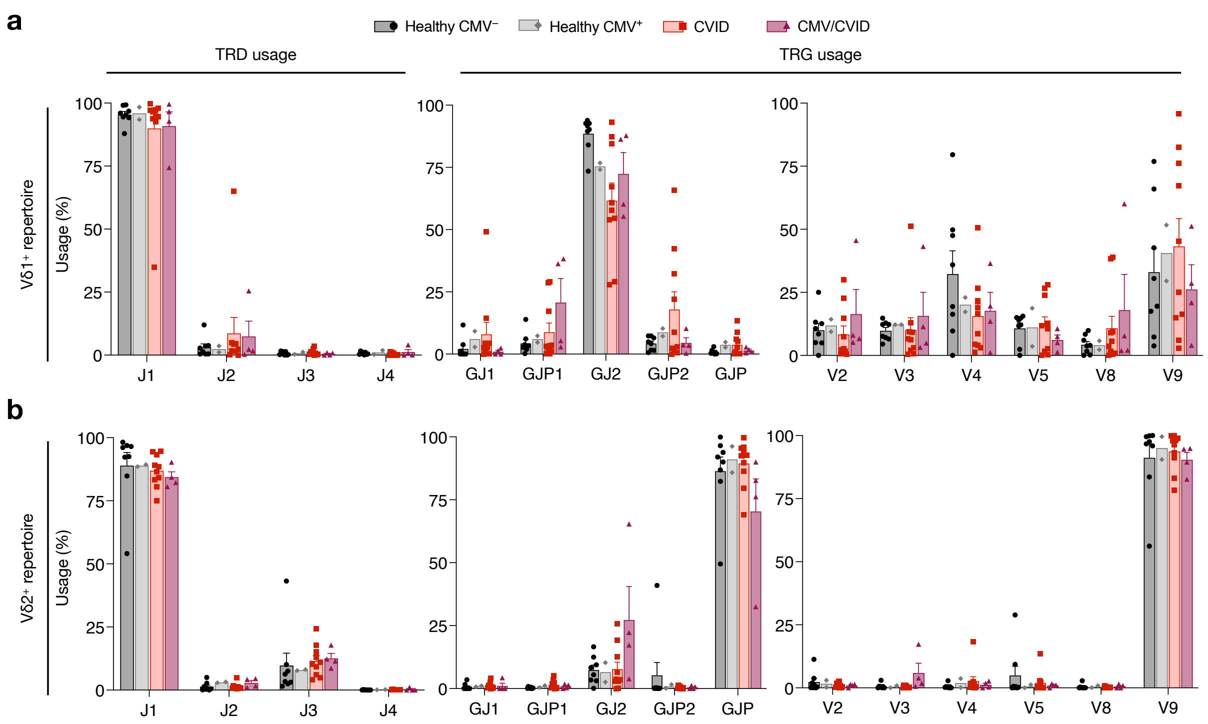

Supplementary Figure 10 – TCRδ chain and TCRγ chain usage for Vδ1^{+} and Vδ2^{+} TCR repertoires. TRD and TRG chain usage shown for (**a**) Vδ1^{+} and (**b**) Vδ2^{+} γδ T cells from healthy CMV^{-} individuals (n = 8) healthy CMV^{+} (n = 2) CVID patients (n = 10), and CMV/CVID patients (n = 4). Each point represents an individual, bar is at mean and error bars are SEM. TRD, T cell receptor delta; TRG, T cell receptor gamma.

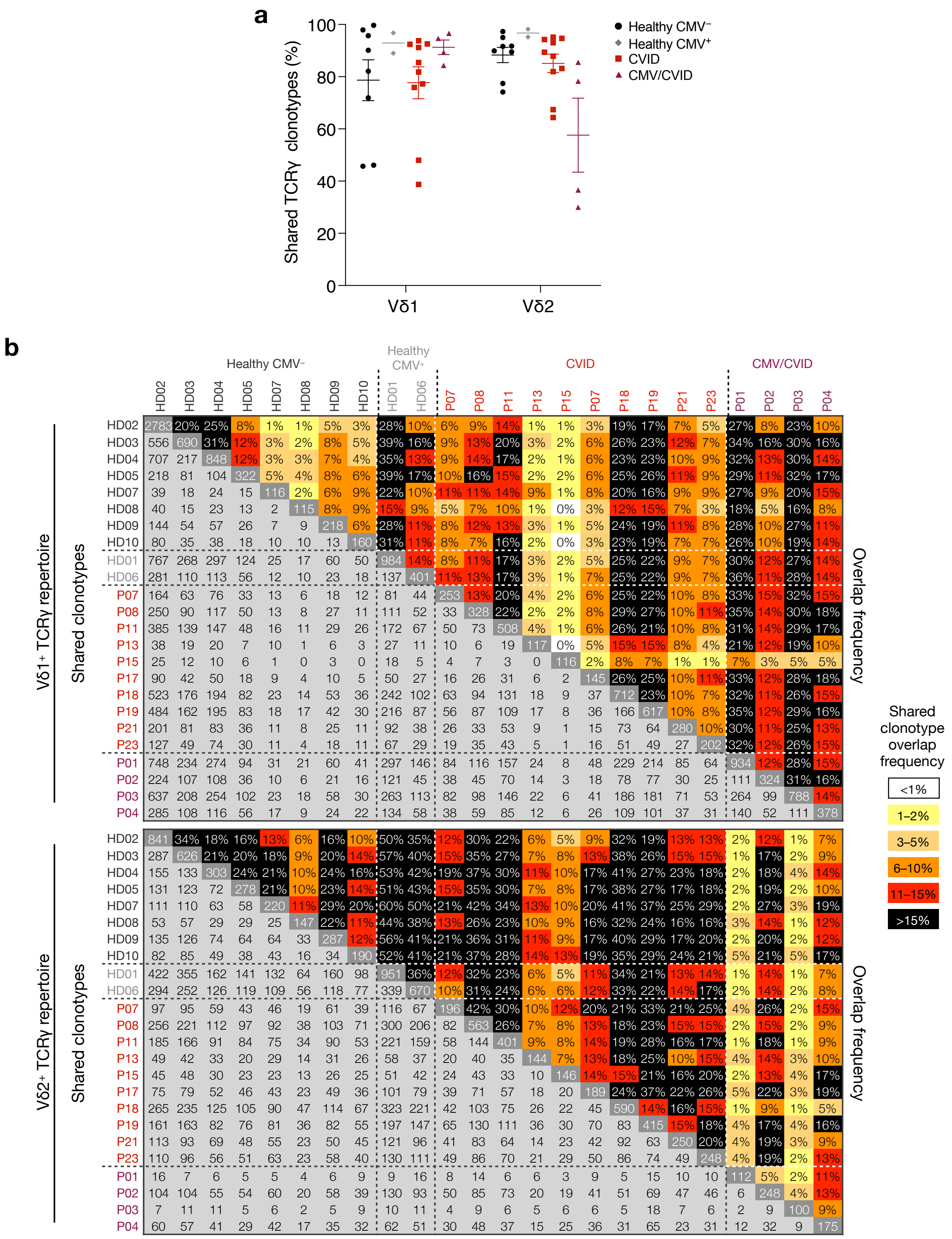

Supplementary Figure 11 – TCRγ repertoire overlap. (**a**) Frequency of shared (present in at least one other sample) CDR3γ (amino acid) sequences from healthy CMV⁻ individuals (n = 8), healthy CMV⁺ individuals (n = 2), CVID patients (n = 10) and CMV/CVID patients (n = 4), where line is mean and error bars represent SEM. (**b**) Quantification (numbers in plots) and frequency (plot colors) of TCRγ clonotypes (abundance, >50 sequencing reads) from healthy CMV⁻ individuals, healthy CMV⁺ individuals, CVID patients and CMV/CVID patients. Plot shows number of shared clonotypes (light gray), frequency of shared clonotypes (color key), and total number of clonotypes for each sample (dark gray diagonal). TCR, T cell receptor.

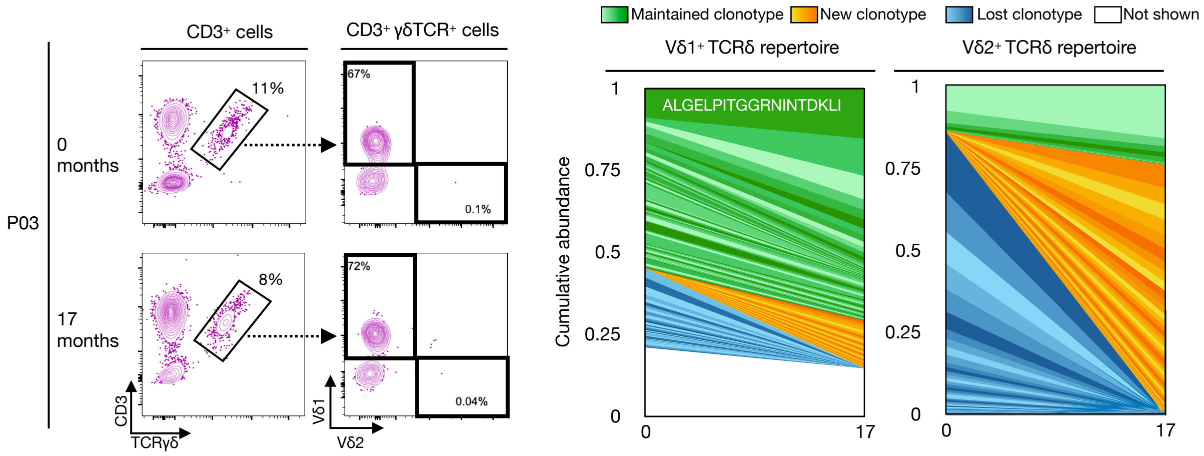

Supplementary Figure 12 – TCRδ clonotype tracking for CMV/CVID patient P03. Plots show gating and frequencies of γδ T cells (as a proportion of $CD3^+$ T cells) and γδ T cell subsets in CMV/CVID patient P03, and corresponding shared clonotype abundance plots for $V\delta1^+$ and $V\delta2^+$ TCRδ repertoire showing the top maintained, new, and lost clones across 17 months. Dominant clonotype TCRδ CDR3 sequence is shown. TCR, T cell receptor.

Supplementary Table 1. CVID patient cohort characteristics.

Donor ID	Sex	Age	Genetic testing	Genetic result	Infectious history	Other complications
**P06**	F	19	Yes	Negative	Recurrent SPI	Nil
**P07**	M	19	No	N/A	Recurrent SPI	Nil
**P08**	M	22	Yes	Negative	Recurrent SPI	Chronic urticaria
**P09**	F	26	Yes	*NFKB2* het P850Sfs36 *	Recurrent SPI	Autoimmune hypothyroidism, isolated ACTH deficiency, ITP, pancreatic islet cell autoimmunity, osteopenia, psoriasis, metabolic syndrome, inflammatory hepatocellular adenoma, minimal change disease
**P10**	F	26	No	N/A	Recurrent SPI	Nil
**P11**	M	32	Yes	Negative	Recurrent SPI, shingles	Osteopenia
**P12**	F	33	Yes	Negative	Recurrent SPI, chronic bilateral mastoiditis, polyomavirus renal tract infection	Enterocolitis, malnutrition, osteoporosis, bronchiectasis, granular CSOM, psoriasiform skin lesions, cystitis, inflammatory sinonasal disease
**P13**	F	37	Yes	*TCF3* het (E555K)	Recurrent SPI	Osteopenia, bronchiectasis, essential thrombocytosis
**P14**	M	41	No	N/A	Recurrent SPI	GLILD, hemangioma
**P15**	F	42	Yes	VUS (*JAK3* het V217M)	Recurrent SPI, pneumonia, gastrointestinal infections	Guillaine-Barre syndrome
**P16**	F	43	No	N/A	Recurrent SPI	Splenic rupture secondary to splenomegaly
**P17**	F	49	Yes	Negative	Recurrent SPI, recurrent urinary tract infections	Alopecia totalis, EPI, thrombocytopenia, malabsorption, chronic diarrhea, non-ischemic cardiomyopathy, congenital valvular heart disease
**P18**	F	55	Yes	Negative	Recurrent SPI, HSV1 keratitis, *Salmonella* gastroenteritis	Ulcerative colitis, psoriasis with inflammatory arthritis
**P19**	F	55	Yes	*NFKB1* het E195Afs*2 9	Recurrent SPI, streptococcal meningitis	ITP, lymphadenopathy
**P20**	F	60	Yes	Negative	Recurrent SPI	Recurrent inflammatory cystitis, osteopenia
**P21**	M	70	No	N/A	Recurrent SPI	Hypospadias
**P22**	F	71	No	N/A	Recurrent SPI	Nil
**P23**	F	75	Yes	VUS (*CR2* het W766X)	Recurrent SPI, fungal infections	Asthma, recurrent rashes

See Table S3 for CMV/CVID cohort details. ACTH, adrenocorticotropic hormone; *CR2*, complement C3d receptor 2; CSOM, chronic suppurative otitis media; EPI, exocrine pancreatic insufficiency; F, female; GLILD, granulomatous-lymphocytic interstitial lung disease; Het, heterozygous; HSV1, herpes simplex virus 1; IGRT, immunoglobulin replacement therapy; IVIg, intravenous immunoglobulin; ITP, immune thrombocytopenic purpura; *JAK3*, janus kinase 3; M, male; N/A, not applicable; *NFKB1*, nuclear factor kappa B subunit 1 gene; ND,

not determined; *NFKB2*, nuclear factor kappa B subunit 2; SCIg, subcutaneous immunoglobulin; *TCF3*, transcription factor 3 gene; SPI, sinopulmonary infection; VUS, variant of uncertain significance.

Supplementary Table 2. Immune T cell counts and frequencies for CVID patients.

**Donor ID**	**Total lymphocyte count** (cells/µL blood)	**T cell count** (cells/µL blood)	**CD4^{+} T cells** (% total T cells)	**CD8^{+} T cells** (% total T cells)	**CD4: CD8 ratio**	**MAIT cells** (% total T cells)	**γδ T cells** (% total T cells)
**P01**	1120	661	48%	28%	1.3	**0.1%**^{L}	**18%**^{H}
**P02**	1380	1007	55%	37%	1.5	**0.3%**^{L}	3.1%
**P03**	**860**^{L}	**447**^{L}	30%	33%	**0.9**^{L}	**0.4%**^{L}	**33%**^{H}
**P04**	**950**^{L}	665	61%	29%	2.1	**0.1%**^{L}	6.2%
**P05**	4090	**3068**^{H}	19%	71%	**0.3**^{L}	**0.05%**^{L}	7.5%
**P06**	3640	**2839**^{H}	51%	39%	1.3	2.7%	5.7%
**P07**	**580**^{L}	**377**^{L}	24%	64%	**0.4**^{L}	1.7%	5.3%
**P08**	**910**^{L}	646	63%	24%	**2.6**^{H}	4.8%	5.2%
**P09**	2100	1302	60%	27%	2.2	3.4%	3.6%
**P10**	1210	871	72%	17%	**4.2**^{H}	5.9%	3.2%
**P11**	**860**^{L}	611	42%	22%	1.9	6.2%	**28%**^{H}
**P12**	1425	1169	78%	17%	**4.6**^{H}	**0.3%**^{L}	2.7%
**P13**	ND	ND	56%	31%	1.8	**0.5%**^{L}	1.6%
**P14**	**830**^{L}	730	42%	32%	1.3	2.8%	**17%**^{H}
**P15**	ND	ND	45%	42%	1.1	**0.8%**^{L}	8.7%
**P16**	2750	1678	45%	42%	1.1	**0.8%**^{L}	2.6%
**P17**	**750**^{L}	**563**^{L}	36%	57%	**0.6**^{L}	**0.3%**^{L}	**0.9%**^{L}
**P18**	2240	1747	32%	52%	**0.6**^{L}	4.9%	7.5%
**P19**	**5030**^{H}	**2716**^{H}	17%	52%	**0.3**^{L}	**0.1%**^{L}	**25%**^{H}
**P20**	ND	ND	53%	37%	1.4	**0.9%**^{L}	3.7%
**P21**	ND	ND	73%	16%	**4.6**^{H}	4.5%	2.7%
**P22**	ND	ND	73%	18%	**4.1**^{H}	6.0%	**0.8%**^{L}
**P23**	ND	ND	67%	23%	**2.9**^{H}	**0.2%**^{L}	1.1%
**Normal reference range**	[1000–4800]	[600–2500]			[1.1-2.4]	1–10%	1–10%

Values in **bold** indicate outside normal healthy range, where ^{H} indicates higher and ^{L} indicates lower than healthy range. MAIT, mucosal associated invariant T (cell); ND, not determined.

Supplementary Table 3. Multivariate analysis of γδ T cell frequencies.

Vδ1 frequency (% γδ T cells)				
**Independent variable**	**β estimate**	**Standard error**	**P value**	**P value summary**
**Diagnosis** [CVID]	15.54	5.42	0.0057	**
**Age**	0.39	0.14	0.0061	**
**CMV viremia** [positive]	47.17	9.79	<0.0001	****
**Sex** [Male]	-1.52	4.81	0.7527	NS

Vδ2 frequency (% γδ T cells)				
**Independent variable**	**β estimate**	**Standard error**	**P value**	**P value summary**
**Diagnosis** [CVID]	8.93	6.07	0.1466	NS
**Age**	0.44	0.16	0.0067	**
**CMV viremia** [positive]	-56.53	10.96	<0.0001	****
**Sex** [Male]	4.58	5.38	0.3979	NS

Statistical analysis performed using multiple linear regression with least squares. NS, not significant.

Supplementary Table 4. CMV/CVID patient cohort details.

	P01	P02	P03	P04	P05
**Age**	62	38	61	60	32
**Sex**	Female	Female	Male	Female	Male
**Clinical diagnosis**	CVID	CVID	CVID	CVID	CVID
**Genetic diagnosis**	*NFKB1* c.730+4A>G het. (splice donor site mutation/skip exon 8)	*NFKB1* c.730+4A>G het. (splice donor site mutation/skip exon 8)	VUS (*ZAP70* c.512A>G, p.E171G het.)	*NFKB1* c.2592+1G>T het. (splice donor site mutation/skip exon 22-predicted)	*NFKB1* c.904dup, p.S302fs het.
**Ig at CVID diagnosis** (g/L)					
IgG [6–16]	**1.3**	**4.7**	**5.8**	**0.7**	**0.5**
IgA [0.8–3]	**<0.04**	**0.4**	**<0.1**	**<0.05**	**<0.1**
IgM [0.4–2.5]	**0.4**	**0.3**	**0.4**	**<0.05**	**0.1**
**Most recent Ig** with IGRT (g/L)					
IgG [6–16]	10.4	7.1	11.7	6.1	7.1
IgA [0.8–3]	**<0.05**	**<0.05**	**0.05**	**<0.05**	**<0.05**
IgM [0.4–2.5]	**<0.05**	**<0.05**	**0.14**	**<0.05**	**<0.05**
**Most recent cell counts** ($\times 10^9$/L blood)					
WCC [4.5–11]	**1.9**	4.6	**1.5**	**2.9**	7.5
Lymphocyte [1–4.8]	1.1	1.5	**0.7**	**0.8**	2.9
$CD19^+$ [0.07–0.55]	**0.01**	**0.01**	**0**	**0.04**	**0**
$CD16^+/56^+$ [0.07–0.7]	**0.04**	0.09	**0**	0.08	0.54
**IGRT**	IVIg (Privigen, 25g, fortnightly) Started 2002	SCIg (Hizentra, 11g, weekly) Started 2015	IVIg (Intragam10, 35g, monthly Started 2011	IVIg (Privigen, 30g, monthly) Started 2016, intermittent use	IVIg (Intragam10, 45g, 3-weekly) Started 2015
**Inflammatory disease**	Autoimmune pancytopenia, NCPH, liver transplant	Chronic diarrhea/ lymphocytic colitis	AIHA, inflammatory colitis, seronegative spondyloarthropathy	ITP	AIHA, inflammatory arthropathy, lymphocytic enteropathy, NCPH
**Other medical history**	Bronchiectasis, malnutrition, osteoporosis	Iron deficiency anemia, chronic sinusitis	Malnutrition, pancreatic insufficiency, T2DM, IHD	Bronchiectasis, pompholyx eczema	Bronchiectasis, corticosteroid myopathy, malnutrition, osteoporosis
**Iatrogenic immuno-suppression** (years administered)	Everolimus + cyclosporin (2016-now); low-dose prednisolone (2016-2021)	Nil	High-dose prednisolone (2012, 2016, 2020, 2022); rituximab (2012, 2020, 2022)	High-dose prednisolone (1998, 2016, 2022, 2023); rituximab (2023)	Rituximab + prednisolone (2017-2019); adalimumab (2021)
**Infectious history**	Chronic norovirus, recurrent *Campylobacter*, recurrent	Recurrent sinopulmonary infections	Oral candidiasis, recurrent gram-negative sepsis (*Salmonella*, *E. coli*,	Recurrent sinopulmonary infections	Chronic *Helicobacter pylori*, pulmonary Aspergillosis,

	sinopulmonary infections		*Pseudomonas*), recurrent sinopulmonary infections		recurrent sinopulmonary infections
**Other medications**	Azithromycin, posoconazole, trimethoprime/sulfamethoxazole, amlodipine, cholecalciferol, moxonidine, pantoprazole, ursodeoxycholic acid, zoledroniec acid	Azithromycin	Trimethoprime/sulfamethoxazole, aspirin, calcium carbonate, insulin, rosuvastatin, pantoprazole, pregabalin	Trimethoprime/sulfamethoxazole	Filgrastim, pantoprazole, vitamin D, folic acid, magnesium, thiamine, ursodeoxycholic acid
**Evidence of tissue invasive CMV disease**	Pneumonitis	Nil	Enteritis, esophagitis	Nil	Enteritis, pneumonitis
**Mode of CMV diagnosis** (year)	BAL PCR (2012)	Whole blood PCR (2021)	IHC and tissue PCR on enteric biopsies (2014, 2020) esophageal biopsy (2022)	Whole blood PCR (2019)	IHC & tissue PCR on enteric biopsy (2018), BAL PCR (2021)
**Age at CMV diagnosis**	52	36	52	56	30
**Peak CMV viral load** (copies/mL)	10 873, log 4.270 (2019)	60, log 1.78 (2021)	614, log 2.79 (2021)	150, log 2.180 (2021)	581 log 4.2 (2019)
**CMV treatment**	Ganciclovir (2016-2018), valganciclovir (2016-2018)	Nil	Ganciclovir (2017, 2020, 2021, 2022), valganciclovir (2017, 2020, 2021, 2022), CMV-TC (2017, 2022)	Nil	Ganciclovir (2018, 2021), valganciclovir (2018, 2021)
**CMV treatment complications**	Pancytopenia	N/A	G-induced neutropenia - resulted in cessation of treatment, then relapsing disease	N/A	Pancytopenia
**CMV Outcome**	Treatment success, chronic asymptomatic viraemia	Monitoring only - stable CMV viral loads	Deceased (2022) - spontaneous atraumatic posterior fossa ICH (ITP likely contributor)	Monitoring only – stable CMV viral loads	Deceased (2021) - overwhelming chest sepsis

[Normal adult reference range]. Values in **bold** indicate outside normal range. AIHA, autoimmune hemolytic anemia; BAL, bronchoalveolar lavage; CMV-TC, Adoptive CMV-specific T lymphocyte therapy; het, heterozygous; ICH, intracerebral hemorrhage; Ig, immunoglobulin; IGRT, immunoglobulin replacement therapy; IHC, immunohistochemistry; IHD, ischemic heart disease; ITP, immune thrombocytopenic purpura; IVIg, intravenous immunoglobulin; N/A, not applicable; NCPH, noncirrhotic portal hypertension; *NFKB1*, nuclear factor kappa B subunit 1 gene; PCR, polymerase chain reaction; SCIg, subcutaneous immunoglobulin; T2DM, type 2 diabetes mellitus; VUS, variant of uncertain significance; WCC, white cell count; *ZAP70*, zeta chain of T cell receptor associated protein kinase 70 gene.

Supplementary Table 5. Vδ1 clonotypic expansions in CMV/CVID patients.

Patient	TCRδ	CDR3	CDR3 length	Frequency in repertoire	Shared or private
P01	Vδ1J1	ALGELDYADWGITYTDKLI	19	28.8%	Private
P02	Vδ1J1	ALGALLLPYYLRGLTSFTDKLI	22	81.7%	Shared
P03	Vδ1J1	ALGELPITGGRNINTDKLI	19	13.5%	Private
P04	Vδ1J1	ALGERIPTVILGDTRWEPAYTDKLI	25	24.1%	Private
	Vδ1J2	ALGGPWFILLGDKTRALTAQLF	22	24.0%	Private
**Patient**	**TCRγ**	**CDR3**	**CDR3 length**	**Frequency in repertoire**	**Shared or private**
P01	Vγ3VJ2	ATWDPYYKKL	10	32.9%	Shared
	Vγ4VJ2	ATWDGPGYKKL	11	6.5%	Shared
P02	Vγ8VJ2	ATWDRDRYYKKL	12	32.6%	Shared
	Vγ8VJP1	ATSPTGWFKI	10	28.9%	Shared
P03	Vγ9VJ2	ALWDHPYYKKL	11	44.8%	Shared
	Vγ9VJ2	ALWEQFYYKKL	11	6.0%	Private
P04	Vγ2VJP1	ATWDGTGWFKI	11	37.2%	Shared
	Vγ9VJ2	ALWEVPGYKKL	11	17.8%	Shared
	Vγ4VJ2	ATWDKDYKKL	10	6.4%	Shared

VJ segments used for TCRδ or TCRγ dominant Vδ1^{+} γδ T cell clonotypes in CMV/CVID patients. CDR3 amino acid sequence, CDR3 length, frequency in total repertoire (first timepoint used for individuals that had two timepoint analysis), and whether the clonotype is a shared (detected in at least one other individual) or private.

Supplementary Table 6. Detailed infectious history between sampling for time course analysis.

	P01	P02	P03
**Date of first sample**	18-03-2020	20-01-2021	24-02-2020
**Date of second sample**	10-05-2023	06-07-2022	21-07-2021
**Iatrogenic immuno-suppression** (years administered)	Everolimus + cyclosporin (2020-2023); low-dose prednisolone (2020-2021)	Nil	High-dose prednisolone and rituximab (2020)
**Infectious history between sampling time course** (date)	*Campylobacter* enteritis (2021)<br>Recurrent infective conjunctivitis (2020-2023)<br>Infective exacerbations of bronchiectasis requiring hospital admission (Jul 2021, Apr & Sept 2022)	Recurrent chest infections while on IGRT<br>Two courses of oral antibiotics (2021-2022)	Dental abscess (Aug 2021)<br>*Salmonella* sepsis (Jun 2021)<br>Polymicrobial sepsis: *E. coli*, *Granulicatella adiacens* bacteremia (Apr 2021)<br>*Pseudomonas* sepsis (Mar 2021)

IGRT, immunoglobulin replacement therapy.

Supplementary Table 7. Number of cells sorted and sequencing reads for TRG and TRD repertoire analysis.

Sample name	Cohort	Cell subset	Frequency (of T cells)	Number of cells sorted	Number of sequencing reads	
					TRG	TRD
**HD01**	Healthy	Vδ1+	1.4%	9 800	224 602	518 551
		Vδ2+	5.4%	36 000	220 960	751 001
**HD02**	Healthy	Vδ1+	0.6%	30 000	372 011	652 578
		Vδ2+	3.1%	100 000	470 059	621 301
**HD03**	Healthy	Vδ1+	0.4%	24 000	85 122	403 986
		Vδ2+	2.3%	76 000	150 737	370 568
**HD04**	Healthy	Vδ1+	0.8%	55 000	473 878	64 174
		Vδ2+	3.5%	100 000	582 540	153 119
**HD05**	Healthy	Vδ1+	0.1%	3 508	292 083	5 972
		Vδ2+	4.3%	100 000	664 324	339 036
**HD06**	Healthy	Vδ1+	1.9%	57 271	546 611	94 028
		Vδ2+	1.9%	58 761	459 893	354 160
**HD07**	Healthy	Vδ1+	0.02%	2 618	85 000	237 413
		Vδ2+	0.8%	18 300	218 125	354 926
**HD08**	Healthy	Vδ1+	0.3%	6 252	654 911	234 046
		Vδ2+	1.2%	22 000	686 672	663 120
**HD09**	Healthy	Vδ1+	4.2%	100 000	691 912	75 005
		Vδ2+	1.8%	39 400	530 870	160 813
**HD10**	Healthy	Vδ1+	2.7%	100 000	872 194	342 761
		Vδ2+	0.2%	10 200	435 305	19 527
**P01_1**	CMV/CVID	Vδ1+	17.1%	100000	178 839	555 165
		Vδ2+	0.08%	529	191 203	534 367
**P01_2**	CMV/CVID	Vδ1+	8.16%	51000	309 191	287 825
		Vδ2+	0.03%	33	377 135	446 252
**P02-1**	CMV/CVID	Vδ1+	2.0%	44 300	572 706	191 943
		Vδ2+	0.1%	2 300	297 121	15 436
**P02-2**	CMV/CVID	Vδ1+	3.8%	69 000	871 167	194 218
		Vδ2+	0.1%	1 700	552 472	31 910
**P03_1**	CMV/CVID	Vδ1+	7.1%	100000	63 307	184 898
		Vδ2+	0.01%	78	94 349	234 441
**P03_2**	CMV/CVID	Vδ1+	5.8%	100000	81 841	177 954
		Vδ2+	0.003%	90	65 597	308 080
**P04**	CMV/CVID	Vδ1+	5.1%	54000	171 478	565 428
		Vδ2+	0.1%	207	334 169	374 332
**P07**	CVID	Vδ1+	1.7%	34000	65 331	156 186
		Vδ2+	1.4%	12600	112 617	282 064
**P08**	CVID	Vδ1+	0.7%	6 610	434 426	415 402
		Vδ2+	4.7%	41 000	528 177	562 802
**P11**	CVID	Vδ1+	0.4%	8 892	518 564	446 230
		Vδ2+	27%	100 000	624 645	738 419
**P13**	CVID	Vδ1+	0.4%	2000	311 427	396 663
		Vδ2+	0.6%	2000	373 451	876 100
**P15**	CVID	Vδ1+	2.5%	1 193	699 750	412 460
		Vδ2+	3.3%	1 433	686 764	552 367
**P17**	CVID	Vδ1+	0.2%	5503	75 945	219 777
		Vδ2+	0.5%	3067	136 440	358 667
**P18**	CVID	Vδ1+	1.4%	56 300	467 100	286 934
		Vδ2+	6.2%	100 000	561 130	334 068
**P19**	CVID	Vδ1+	10.7%	100000	263 722	626 137
		Vδ2+	0.45%	13000	320 132	686 790

**P21**	CVID	Vδ1$^+$	2.1%	20 800	792 323	23 379
		Vδ2$^+$	0.7%	8 403	674 033	34 523
**P23**	CVID	Vδ1$^+$	0.5%	9 140	450 574	76 831
		Vδ2$^+$	0.4%	7 924	253 778	52 228

TRD, T cell receptor delta; TRG, T cell receptor gamma.
